# Latrunculin A-Induced Perturbation of the Actin Cytoskeleton Mediates Pap1p-Dependent Induction of the Caf5p Efflux Pump in *Schizosaccharomyces pombe*

**DOI:** 10.1534/g3.116.037903

**Published:** 2016-12-29

**Authors:** Farzad Asadi, Bidhan Chakraborty, Jim Karagiannis

**Affiliations:** Department of Biology, The University of Western Ontario, London, Ontario N6A 5B7, Canada

**Keywords:** fission yeast, latrunculin A, cell division, actin cytoskeleton, stress response

## Abstract

As part of an earlier study aimed at uncovering gene products with roles in defending against latrunculin A (LatA)-induced cytoskeletal perturbations, we identified three members of the oxidative stress response pathway: the Pap1p AP-1-like transcription factor, the Imp1p α-importin, and the Caf5p efflux pump. In this report, we characterize the pathway further and show that Pap1p translocates from the cytoplasm to the nucleus in an Imp1p-dependent manner upon LatA treatment. Moreover, preventing this translocation, through the addition of a nuclear export signal (NES), confers the same characteristic LatA-sensitive phenotype exhibited by *pap1*Δ cells. Lastly, we show that the *caf5* gene is induced upon exposure to LatA and that Pap1p is required for this transcriptional upregulation. Importantly, the expression of *trr1*, a Pap1p target specifically induced in response to oxidative stress, is not significantly altered by LatA treatment. Taken together, these results suggest a model in which LatA-mediated cytoskeletal perturbations are sensed, triggering the Imp1p-dependent translocation of Pap1p to the nucleus and the induction of the *caf5* gene (independently of oxidative stress).

The ability to adapt to external stresses requires the rapid activation of transcriptional programs that promote cell survival. These programs are dependent upon the action of transcription factors that must properly decode upstream signals to affect the required changes in gene expression. In the fission yeast *Schizosaccharomyces pombe* the product of the *pap1* gene is key in a variety of such transcriptional responses (*e.g.*, oxidative stress, nitrosative stress, methylglyoxal stress, the DNA damage response, and drug and heavy metal stress) (Toone *et al.* 1998; Chen *et al.* 2003; Zuin *et al.* 2005; Belfield *et al.* 2014; Biswas and Ghosh 2015).

The *pap1* gene encodes a bZIP domain containing a transcription factor that shares similarity with the mammalian c-Jun protein (a component of the AP-1 transcription factor complex involved in cell growth, differentiation, and apoptosis) (Toone *et al.* 1998; Eferl and Wagner 2003). *pap1* was first isolated and characterized in a screen for genes that, when overexpressed, conferred resistance to staurosporine (Toda *et al.* 1991). Subsequent work revealed that increased *pap1* levels provided resistance, not only to staurosporine but also to a variety of different drugs (Turi *et al.* 1994; Arioka *et al.* 1998). Conversely, *pap1* loss of function mutants were shown to be hypersensitive to a multitude of toxic compounds, including anisomycin, arsenic, cadmium, camptothecin, and cycloheximide, to name just a few (Toone *et al.* 1998; Han *et al.* 2010). This involvement in multidrug resistance is mediated, at least in part, through Pap1p-dependent regulation of the Bfr1p, Pmd1p, and Caf5p efflux pumps (Toone *et al.* 1998; Calvo *et al.* 2009; Kawashima *et al.* 2012).

In addition to regulating multidrug resistance, *pap1* has a clear and well-defined role in the cellular response to oxidative stress. When challenged with oxidants that lead to abnormally high intracellular levels of reactive oxygen species (ROS), Pap1p activates a group of 50–80 genes (encoding both antioxidant enzymes and efflux pumps) that play a role in defending against oxidative damage (Toone *et al.* 1998; Chen *et al.* 2008; Calvo *et al.* 2013). Interestingly, this ROS-dependent induction depends on the regulated translocation of Pap1p from the cytoplasm to the nucleus. This translocation is in turn influenced by the redox-sensitive formation of a disulfide bond between two cysteine residues in the protein (C278 and C501). Formation of the bond is thought to inhibit the function of a NES found within Pap1p, thereby preventing its association with the nuclear export machinery. This results in the accumulation of Pap1p in the nucleus until the redox state of the cell returns to normal (Kudo *et al.* 1999; Castillo *et al.* 2002; Calvo *et al.* 2013).

In addition to nuclear export, Pap1p localization is also controlled at the level of nuclear import. Pap1p contains two overlapping bipartite-type nuclear localization sequences (NLSs) that mediate its interaction with the α-importin, Imp1p. This interaction is necessary for translocation since, unlike wild-type cells, *imp1*Δ mutants fail to accumulate Pap1p in the nucleus when exposed to oxidative stress (Umeda *et al.* 2005). Importantly, while Pap1p must be oxidized to upregulate the transcription of antioxidant genes (*e.g.*, *trr1*), constitutively reduced, nuclear-localized Pap1p is fully capable of upregulating the transcription of drug resistance genes (*e.g.*, *caf5*). Thus, the role of Pap1p in transcriptional regulation is genetically separable into at least two distinct pathways (Calvo *et al.* 2012).

In this report, we characterize *pap1*, *imp1*, and *caf5* with respect to their role in defending against abnormal cytoskeletal perturbations. This role was uncovered during the course of a genome-wide screen for gene deletion mutants displaying hypersensitivity to the actin depolymerizing drug LatA (Asadi *et al.* 2016). Contrary to a simple model in which Pap1p constitutively regulates the basal levels of Caf5p expression, we show that Pap1p translocates from the cytoplasm to the nucleus in an Imp1p-dependent manner upon LatA-induced cytoskeletal stress. Furthermore, we show that Pap1p itself is required for the LatA-dependent induction of the Caf5p efflux pump. Significantly, the expression of *trr1*, an oxidative stress-specific Pap1p target, is not affected by LatA treatment. These results suggest a model in which LatA-mediated cytoskeletal perturbations are sensed, leading to the nuclear translocation of reduced Pap1p and the exclusive activation of the multidrug resistance arm of the pathway.

## Materials and Methods

### Yeast methods

*S. pombe* cells were cultured in YES or EMM media supplemented with adenine, histidine, leucine, and/or uracil (Forsburg and Rhind 2006). Liquid cultures were grown with shaking (200 rpm) at 30°. In experiments involving LatA treatment, *S. pombe* cells were grown to midlog phase (O.D. of 0.2) in YES and treated with the indicated concentration of LatA (Enzo Life Sciences International). Cells were then grown at 30° for the indicated duration before being fixed with ethanol and stored in PBS pH 7.4.

In experiments involving hydrogen peroxide treatment, *S. pombe* cells were grown to midlog phase (O.D. of 0.2) in YES and then treated with 0.003% hydrogen peroxide (Sigma). Cells were grown at 30° for the indicated duration before being analyzed further.

All strains used in this study were either derived from the Karagiannis lab collection, constructed during the course of this work (see *Cloning methods*), or in the case of the *imp1*Δ and *caf5*Δ strains, purchased from Bioneer Corporation as part of version 4 of the haploid gene deletion mutant library (Kim *et al.* 2010). All genetic crosses and general yeast techniques were performed using standard methods (Forsburg and Rhind 2006)

### Spot assays

Strains of the indicated genotype were grown overnight at 30° in liquid YES media to an O.D. of 0.5. Five microliters of undiluted culture, as well as four ten-fold serial dilutions (made in liquid YES), were then spotted onto YES-agar plates containing DMSO (solvent control), or 0.1, 0.2, or 0.3 µM LatA. Growth was assayed visually after the plates had been incubated for 4 d at 30°.

### Cloning methods

To construct the *pap1*∆ deletion mutant, a PCR-based cloning method based on the method of Gregan *et al.* (2006) was used. A 606 bp sequence located upstream of the *pap1* open reading frame, and a 285 bp sequence located downstream of *pap1*, were PCR amplified using primer sequences obtained from http://mendel.imp.ac.at/Pombe_deletion/. The primers used for the upstream region incorporated *Nhe*I and *Ssp*BI restriction sites. The primers used for the downstream region incorporated *Nhe*I and *Bam*HI restriction sites. Both the PCR products were digested with *Nhe*I and subsequently ligated. The ligated PCR product was then double-digested with *Ssp*BI and *Bam*HI and cloned into the pCloneHYG1 vector. The vector was then linearized with *Nhe*I and transformed into *S. pombe*. Hygromycin-resistant transformants were then isolated and subjected to colony PCR, using primer sequences obtained from http://mendel.imp.ac.at/Pombe_deletion/, to identify clones in which the linear dsDNA fragment had replaced the endogenous *pap1* gene via homologous recombination.

The pap1-GFP integrant strain was created using a PCR-based cloning method. First, a C-terminal fragment of the *pap1* gene was PCR amplified from genomic DNA (forward: 5′-GGC GGG AAT TCT CTA ACG AAA ATG GA-3′, reverse: 5′-GGC GGC CCG GGA TTA AAT TGA TTT AA-3′). Following PCR amplification, the amplicon was cloned into the *Eco*RI and *Sma*I sites of the pJK210‐GFP vector. Finally, the plasmid was transformed into a *ura4-D18 S. pombe* strain using the lithium acetate method (Forsburg and Rhind 2006). Ura^+^ transformants were then selected using EMM-agar media lacking uracil. The Ura^+^ transformants were verified for locus-specific homologous recombination by colony PCR (forward: 5′-GGC GGG AAT TCT CTA ACG AAA ATG GA-3′, reverse: 5′-TGG GAC AAC TCC AGT GAA AA-3′). The Pap1-NES-GFP strain was made in the same way using reverse primers incorporating an NES sequence (NESreverse: 5′-GGC GGC CCG GGG TCT AGG GTG AGA CGT TCT AGC GGG AGC TGT AGA TTA AAT TGA TTT AA-3′).

### Fluorescence microscopy

To observe nuclei and cell wall/septal material, cells were fixed with two volumes of ice-cold ethanol and then spun at 5000 rpm for 2 min. The pellet was then resuspended in 1 ml of PBS (pH 7.4) containing 1% Triton X-100. Cells were subsequently washed three times in PBS before being resuspended in 100 µl of PBS containing 15% glycerol. Cells were then mixed with 0.02 mg/ml 4′6-diamidino-2-phenylindole (DAPI) and 1 mg/ml aniline blue. Fluorescence images (DAPI filter set) were obtained with a Zeiss Axioskop two microscope attached to a Scion CFW Monochrome CCD Firewire Camera (Scion Corporation). The microscope system was driven by ImageJ 1.41 software (National Institutes of Health). *S. pombe* cells expressing Pap1-GFP fusions were observed live (GFP filter set) using a Leica DMI6000B inverted microscope equipped with a 100 × Plan Apochromat 1.4 NA oil objective and a Photometrics QuantEM:512SC EMCCD camera driven by Metamorph software.

### qRT-PCR

Total RNA was isolated using TRIzol Reagent (Life Technologies) according to the supplier’s protocol. cDNA synthesis was performed with 1 µg of total RNA using the SuperScript III First Strand Synthesis Supermix for qRT-PCR kit (Invitrogen), according to the supplier’s protocol. Real-time PCR was performed using a Bio-Rad CFX Connect Real-Time PCR Detection System in conjunction with the Maxima SYBR Green qPCR Master Mix kit (Thermo Scientific). Primers specific for *caf5* (forward: 5′-CTC AGC TTG CAA AGG AAA CC-3′, reverse: 5′-GGC ACC CAC GAG AAT ACC TA-3′), *trr1* (forward: 5′-TCT TTC TCG CCC TTT CA-3′, reverse: 5′-GGC ACC ATC ACA TAC AGC AC-3′), and the control housekeeping gene *gpd1* (forward: 5′-TCT GCC GGT ATC CAA CTT TC-3′, reverse: 5′-CAC TGC AAA CGA CAA CGA CT-3′) were used. Relative expression levels were determined using the 2^−ΔΔCt^ method of Livak and Schmittgen (2001). Statistical analysis was performed using one-way ANOVA.

### Data availability

The authors state that all data necessary for confirming the conclusions presented in the article are represented fully within the article.

## Results

### pap1, imp1, and caf5 gene deletion mutants are unable to complete cytokinesis in the presence of low doses of the actin depolymerizing drug, latrunculin A

As part of a broader study examining the effects of LatA-induced cytoskeletal perturbations, we identified a small group of *S. pombe* gene deletion mutants that are hypersensitive to the drug (Asadi *et al.* 2016). Inspection of the LatA-sensitive list revealed the presence of three genes (*pap1*, *imp1*, and *caf5*) that had been previously characterized, and which are known to function together as part of a regulatory module that protects *S. pombe* cells against oxidative stress (Umeda *et al.* 2005). We thus reasoned that this same module might also function independently of oxidative stress (and perhaps using alternate effectors) to counter the detrimental effects of LatA treatment.

**Figure 1:**
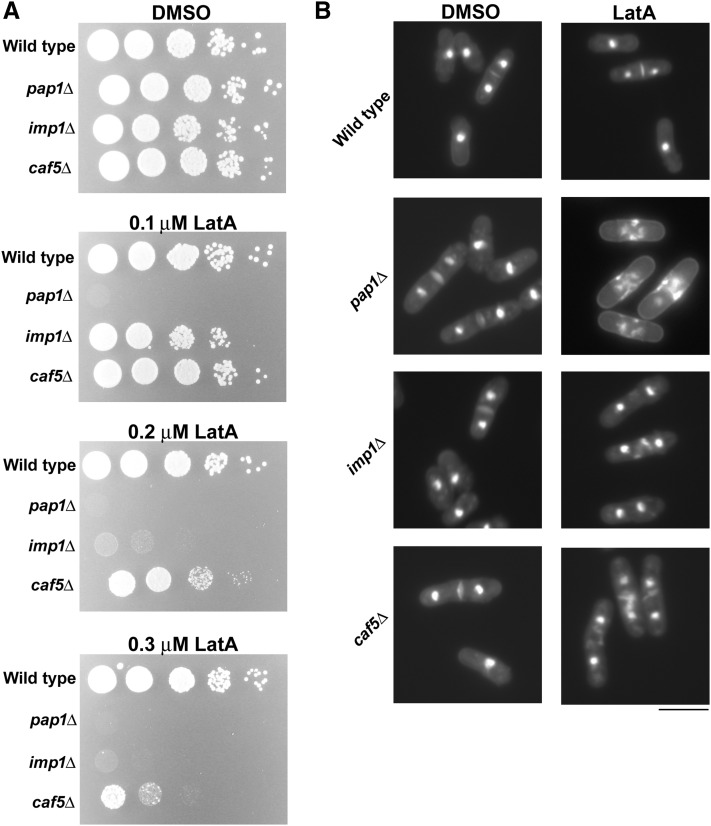
*pap1*, *imp1*, and *caf5* gene deletion mutants are unable to proliferate on media containing low doses of the actin depolymerizing drug LatA. (A) Serial dilutions (10-fold) of cultures of the indicated genotypes were spotted onto YES-agar plates containing DMSO (solvent control), or 0.1, 0.2, or 0.3 μM LatA. Photographs were taken after 4 d incubation at 30°. (B) Cells of the indicated genotype were grown to midlog phase and then treated with DMSO (solvent control) or 0.3 μM LatA for 5 hr. Cells were fixed and stained with DAPI/aniline blue to visualize nuclei and cell/wall septal material, respectively. Scale bar, 10 μm. DAPI, 4′6-diamidino-2-phenylindole; DMSO, dimethyl sulfoxide; LatA, latrunculin A; YES, yeast extract sucrose.

We began our analysis by validating the results of the initial screen. This was done by culturing the respective gene deletion mutants in liquid YES media and then spotting 10-fold serial dilutions of the log-phase cultures onto YES-agar plates containing DMSO (solvent control) or LatA. While wild-type control cells were viable, and grew even at 0.3 µM LatA, *pap1*Δ cells were unable to proliferate at concentrations as low as 0.1 µM LatA. In contrast, *imp1*Δ cells grew at 0.1 µM LatA, but not at 0.2 or 0.3 µM LatA. Lastly, *caf5*Δ cells were the least sensitive, being able to proliferate at 0.1 and 0.2 µM LatA, but not at 0.3 µM LatA (Figure 1A).

Having validated the hits of the initial screen, we were next interested in identifying the root cause of the mutants’ sensitivity to LatA. To explore this question, we grew the mutants to midlog phase in liquid YES and treated them with 0.3 µM LatA for 5 hr. The samples were then fixed with ethanol and stained with a mixture of DAPI and aniline blue (to visualize nuclei and cell wall/septal material, respectively). While all three mutants grew normally in YES media containing DMSO, they were unable to divide successfully in the presence of LatA. Instead, the mutant samples accumulated a high percentage of binucleate cells with fragmented septa (Figure 1B and Table 1). Wild-type cells on the other hand, while sometimes forming malformed septa, were for the most part able to divide successfully. These data demonstrate that the *pap1*, *imp1*, and *caf5* genes are required to mitigate the detrimental effects of LatA exposure and that, in their absence, *S. pombe* cells fail in cytokinesis.

**Table 1:** Mean percentage of cells (± SD) displaying the indicated phenotype after 5 hr treatment with 0.3 µM latrunculin A (*n*  =  3)

Genotype	Uninucleate	Binucleate (Complete Septum)	Binucleate (Fragmented Septum)	Tetranucleate (Fragmented Septum)	Ratio (Fragmented/Nonfragmented)
Wild type	78 ± 15	14 ± 12	8 ± 2	0	0.09
*pap1*Δ	43 ± 5	1 ± 1	56 ± 5	0	1.29
*imp1*Δ	32 ± 19	1 ± 1	64 ± 17	1 ± 1	1.95
*caf5*Δ	37 ± 5	2 ± 2	61 ± 6	0	1.56
*pap1-NES-GFP*	21 ± 7	2 ± 2	71 ± 9	6	3.29
*act1-R183A*,*D184A*	58 ± 4	33 ± 5	9 ± 3	0	0.10

### Pap1p translocates from the cytoplasm to the nucleus upon LatA treatment

Previous studies concerning the role of *pap1*, *imp1*, and *caf5* in the oxidative stress response (Umeda *et al.* 2005) suggested a straightforward model to be tested. In this model, the Pap1p transcription factor is imported into the nucleus via the α-importin, Imp1p. Once present in the nucleus, Pap1p acts to induce the expression of the *caf5* efflux pump, thereby leading to the export of intracellular LatA. In one version of the model, Pap1p acts as a constitutive, positive regulator of *caf5* transcription (*i.e.*, Pap1p performs this function as a matter of course, in the absence of any specific initiating signal). In this scenario, the basal levels of *caf5* expression are simply lower in *pap1*Δ backgrounds, leading to the observed sensitivity of *pap1*Δ cells to LatA.

In another version of the model, LatA-induced cytoskeletal damage acts as the initiating signal. This is to say, the presence of intracellular LatA (or some LatA-induced defect) leads to the activation of the module and the *ad hoc* accumulation of Pap1p in the nucleus. Once accumulated to sufficient levels, Pap1p then induces the expression of the *caf5* gene in order to promote LatA efflux.

To explore these two versions of the model, we constructed a C-terminal Pap1-GFP strain in which the fusion protein was expressed under the control of the native *pap1* promoter (see *Materials and Methods*). The strain appeared phenotypically normal and exhibited levels of LatA sensitivity that were indistinguishable from that shown by a wild-type strain (data not shown). This demonstrated that the addition of the GFP tag had not negatively impacted the function of the protein (at least with respect to its role in responding to LatA). While many previous studies have employed an N-terminal GFP-pap1 fusion, it should be noted that this construct places *pap1* under the control of the nonnative *nmt41* promoter and results in the overexpression of the protein relative to the fusion employed in this study.

**Figure 2:**
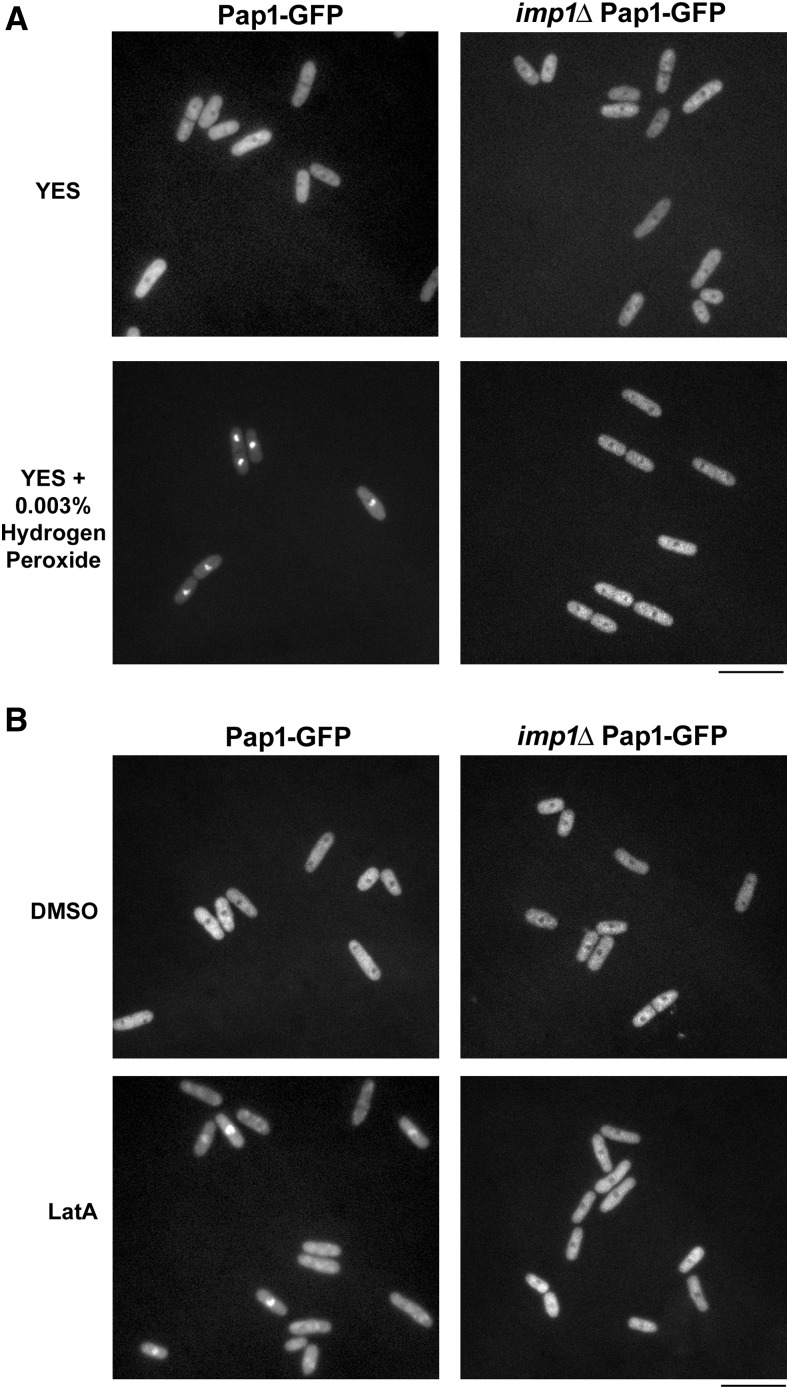
Pap1p translocates from the cytoplasm to the nucleus in an *imp1*-dependent manner upon LatA treatment. (A) Pap1-GFP expressing cells of the indicated genotype were grown to midlog phase and then treated with 0.003% hydrogen peroxide. Cells were imaged 15 min post-treatment using the GFP filter set. (B) Pap1-GFP expressing cells of the indicated genotype were grown to midlog phase and then treated with DMSO (solvent control) or 0.3 μM LatA. Cells were imaged 30 min post-treatment using the GFP filter set. Scale bar, 20 μm. DMSO, dimethyl sulfoxide; GFP, green fluorescent protein; LatA, latrunculin A; YES, yeast extract sucrose.

Next, to ensure that we could detect nuclear localized Pap1-GFP, we cultured the Pap1-GFP-expressing strain to midlog phase and treated the cells with 0.003% hydrogen peroxide. As expected, Pap1-GFP could be detected in the nucleus within 15 min of hydrogen peroxide exposure, but was excluded from the nucleus in the absence of treatment (Figure 2A).

We proceeded by culturing the Pap1-GFP strain to midlog phase and treating the cells with either DMSO (solvent control) or with 0.3 µM LatA. As expected, Pap1-GFP in DMSO-treated control cells was found to localize almost exclusively in the cytoplasm. In contrast, Pap1-GFP signal in LatA-treated cells was enriched in the nucleus (Figure 2B). However, it should be noted, that the strength of the response to LatA seemed weaker than that seen for hydrogen peroxide treatment. While the hydrogen peroxide-treated samples displayed strong nuclear signals in almost all cells, the LatA-treated strain exhibited a much more heterogeneous response. The nuclear GFP signal ranged from being approximately equal to that seen in the cytoplasm, to being strongly enriched in the nucleus relative to the cytoplasm. In any event, the observed LatA-dependent accumulation of Pap1-GFP in the nucleus does not support a simple model in which Pap1p constitutively regulates the basal levels of Caf5p expression. Instead, the data support a scenario in which Pap1p actively responds to LatA-induced cytoskeletal perturbations by translocating into the nucleus.

We next examined whether the α-importin Imp1p was involved in Pap1p nuclear translocation. In strong contrast to *imp1*^+^ cells, Pap1-GFP in *imp1*Δ mutants was found exclusively in the cytoplasm after treatment with LatA at concentrations ranging from 0.1 to 0.3 µM (Figure 2B, data not shown). Thus, similar to what is seen in response to oxidative stress, the observed LatA-induced translocation of Pap1p is Imp1p-dependent.

### Preventing Pap1p translocation to the nucleus confers LatA sensitivity

Our previous data suggested that Pap1-GFP nuclear translocation was a key event in the cells’ response to LatA-induced cytoskeletal stress. To determine if the observed translocation was indeed physiologically relevant, we constructed a Pap1-GFP strain that incorporated an exogenous nuclear export sequence (Pap1-NES-GFP) to prevent Pap1p accumulating in the nucleus. As expected, the Pap1-NES-GFP strain exhibited predominantly cytoplasmic localization in both the presence and absence of LatA (Supplemental Material, Figure S1).

**Figure 3:**
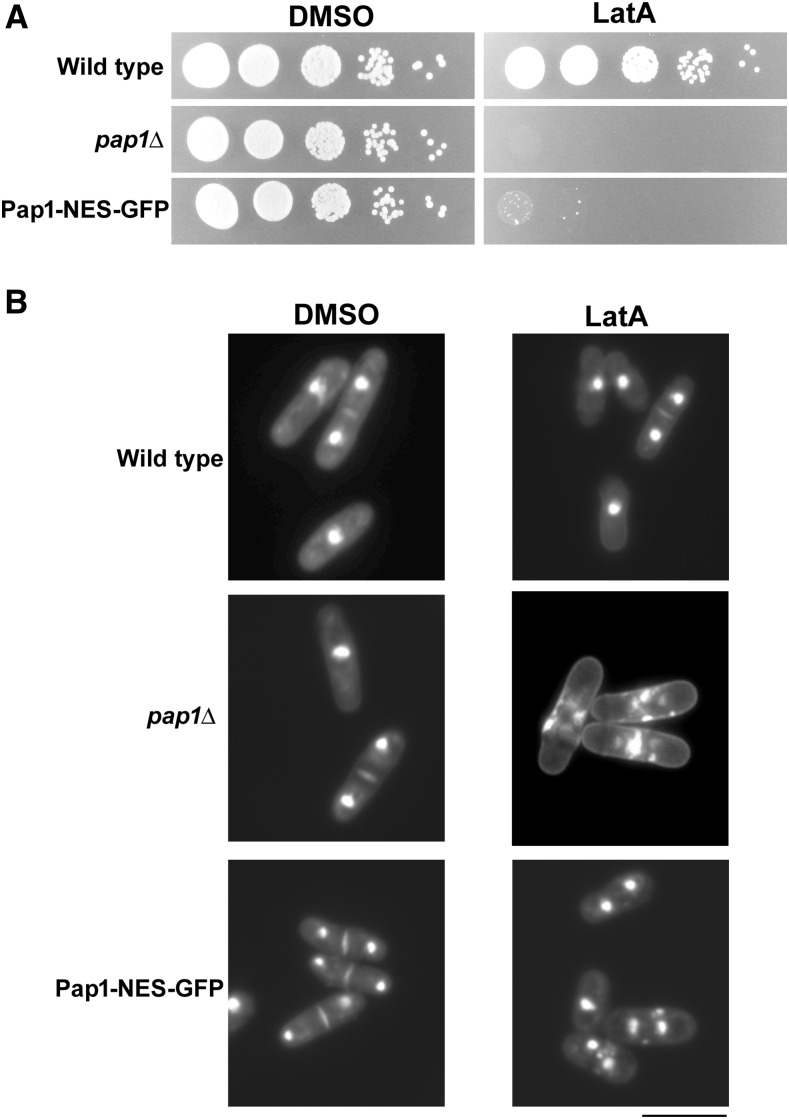
Cells expressing Pap1-NLS-GFP are unable to proliferate on media containing low doses of the actin depolymerizing drug LatA. (A) Tenfold serial dilutions of cultures of the indicated genotypes were spotted onto YES-agar plates containing DMSO (solvent control) or 0.3 μM LatA. Photographs were taken after 4 d incubation at 30°. (B) Cells of the indicated genotype were grown to midlog phase and then treated with DMSO (solvent control) or 0.3 μM LatA for 5 hr. Cells were fixed and stained with DAPI/aniline blue to visualize nuclei and cell/wall septal material, respectively. Scale bar, 10 μm. DAPI, 4′6-diamidino-2-phenylindole; DMSO, dimethyl sulfoxide; GFP, green fluorescent protein; LatA, latrunculin A; NLS, nuclear localization sequence; YES, yeast extract sucrose.

To determine the effect of Pap1p mislocalization on LatA sensitivity, we first spotted cells from log-growing cultures of wild-type, *pap1*Δ, and Pap1-NES-GFP strains onto YES media containing DMSO or LatA. As expected, all three strains were capable of growth on YES-DMSO plates. In contrast, while the wild-type strain was indeed viable in the presence of LatA, both the *pap1*Δ and the Pap1-NES-GFP strains were incapable of forming colonies (Figure 3A). Furthermore, when strains were examined with DAPI/aniline blue, the pap1-NES-GFP strain, similarly to the *pap1*Δ control, accumulated a high proportion of cells that failed in cytokinesis (Figure 3B and Table 1). These data clearly demonstrate that Pap1p translocation into the nucleus is a necessary event in the cellular response to LatA-mediated cytoskeletal perturbation.

### Pap1p fails to translocate in response to LatA in act1-R183A,D184A mutant backgrounds

We were next interested in better characterizing the nature of the signal that led to Pap1p nuclear accumulation. One possibility was that changes to the actin cytoskeleton itself were being sensed. Alternatively, the mere presence of the foreign LatA molecule (or perhaps an actin-independent process related to intracellular LatA concentration) could be triggering Pap1p translocation. To distinguish between these two possibilities, we made use of an actin allele (*act1-R183A*,*D184A*) that is insensitive to the depolymerizing effects of LatA (Ayscough *et al.* 1997; Fujita *et al.* 2003; Karagiannis *et al.* 2005). This allele contains two transition mutations (R183A and D184A) that confer resistance to LatA by affecting the LatA binding site (Ayscough *et al.* 1997). Remarkably, fission yeast cells carrying the allele are able to grow, with no ill effects, at concentrations of the drug that are lethal to wild type (Figure S2).

**Figure 4:**
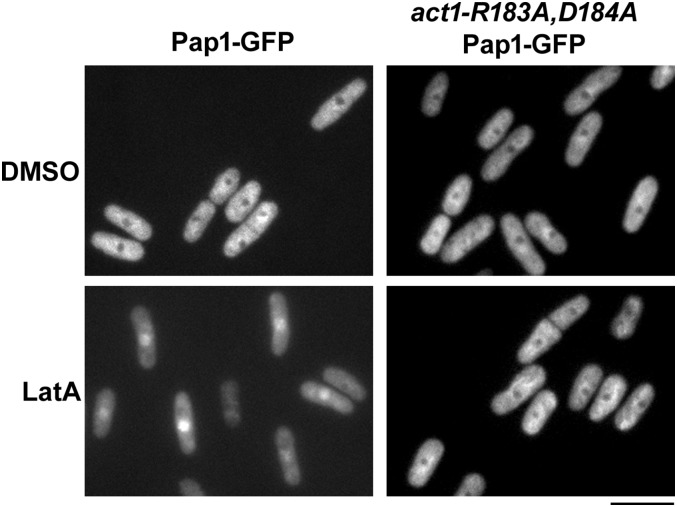
Pap1p fails to translocate in response to LatA in *act1-R183A*,*D184A* mutant backgrounds. Pap1-GFP-expressing cells of the indicated genotype were grown to midlog phase and then treated with DMSO (solvent control) or 0.3 μM LatA. Cells were imaged 30 min post-treatment using the GFP filter set. Scale bar, 10 μm. DMSO, dimethyl sulfoxide; GFP, green fluorescent protein; LatA, latrunculin A.

If *S. pombe* cells were sensing the presence of LatA itself (or an actin-independent process affected by LatA) then one would expect to see translocation of Pap1p from the cytoplasm to the nucleus in an *act1-R183A-D184A* Pap1-GFP strain. Alternatively, if the cell was sensing perturbation of the actin cytoskeleton, then one would expect Pap1p to remain in the cytoplasm upon LatA treatment. While we observed translocation of Pap1-GFP upon LatA treatment in wild-type controls, Pap1-GFP remained in the cytoplasm in an *act1-R183A-D184A* background (Figure 4). This result strongly implies that cytoskeletal perturbation resulting from the binding of LatA to actin, as opposed to an actin-independent process related to intracellular LatA levels, signals Pap1p nuclear accumulation.

### The caf5 gene is induced in a pap1-dependent manner in response to LatA treatment

Another key prediction of our model concerns the relationship of *pap1* to the multidrug resistance and antioxidant arms of the pathway. If correct in our thinking, we would expect Pap1p to act as a positive regulator of *caf5* (multidrug resistance arm), but not *trr1* (antioxidant arm), upon LatA treatment. To determine if this was the case, we used qRT-PCR to assay the levels of *caf5* and *trr1* transcripts in the presence or absence of LatA.

**Figure 5:**
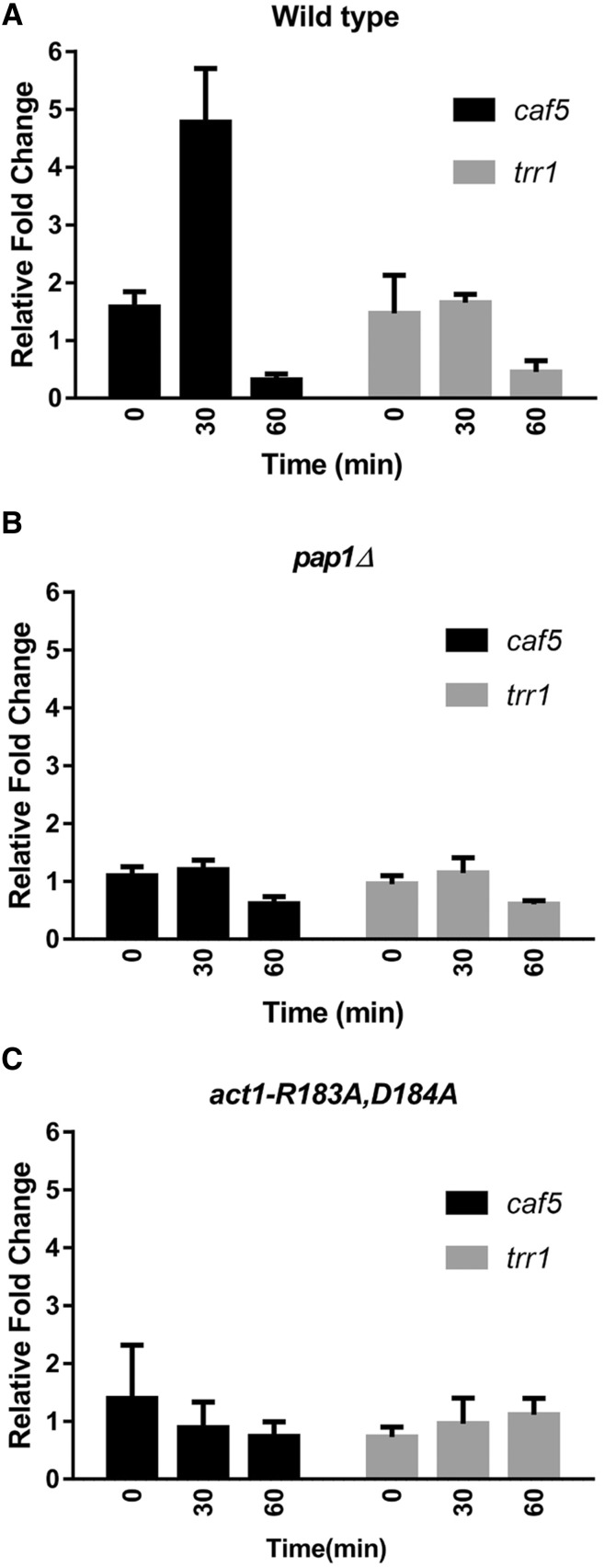
The *caf5* gene is induced in a *pap1*-dependent manner in response to LatA. qRT-PCR analysis of *caf5* and *trr1* expression (relative to *gpd1*) in response to 0.3 μM LatA treatment in (A) wild-type, (B) *pap1*Δ, and (C) *act1-R183A*,*D184A* backgrounds. The level of *caf5* and *trr1* transcripts were normalized to that of the internal control *gpd1*. The normalized level of the transcripts in LatA-treated cells is shown relative to that of DMSO controls. The mean ± SE for three biological replicates is shown. DMSO, dimethyl sulfoxide; LatA, latrunculin A; qRT-PCR, quantitative real time-polymerase chain reaction.

As expected, we observed a ∼4.5-fold induction of the *caf5* gene 30 min after LatA treatment in a wild-type background (*P* = 0.007) (Figure 5A). In contrast, *pap1*Δ cells showed only a ∼1.1-fold induction of *caf5* (*P* = 0.63) (Figure 5B). The difference in mean *caf5* expression level between wild-type and *pap1*Δ strains after 30 min LatA treatment was significant (*P* = 0.02). Thus, *caf5* induction is indeed Pap1p-dependent. Interestingly, two other Pap1p-dependent, hydrogen peroxide-induced efflux pumps (*bfr1* and *pmd1*) were not induced by LatA treatment (data not shown). This is consistent with the fact that neither *bfr1*Δ or *pmd1*Δ were isolated as LatA-sensitive hits in our original screen (Asadi *et al.* 2016).

Significantly, while we observed a strong induction of both *caf5* (*P* = 0.003) and *trr1* (*P* = 0.005) transcripts upon hydrogen peroxide treatment (Figure S3), we did not observe any significant change in *trr1* transcript levels upon exposure to LatA at any time point (Figure 5). Two other oxidative stress-induced transcripts (*ctt1* and *trx2*) also did not show any significant changes in expression upon LatA treatment (data not shown). Thus, consistent with our model, these data indicate that only the multidrug resistance arm of the pathway is activated upon LatA treatment. Importantly, we also did not observe any significant *caf5* induction in *act1-R183A*,*D184A* backgrounds (*P* = 0.62) (Figure 5C). Thus, consistent with our previous findings, this result implies that Pap1p-dependent *caf5* induction is signaled by perturbation of the actin cytoskeleton itself.

## Discussion

While the Pap1p transcription factor clearly plays an important role in the oxidative stress response of fission yeast cells (Toone *et al.* 1998; Chen *et al.* 2003), more recent work suggests that it also functions in a variety of other stress response pathways (Zuin *et al.* 2005; Belfield *et al.* 2014; Biswas and Ghosh 2015). For example, *pap1* becomes essential when cells are challenged with methylglyoxal, a toxic metabolite generated during glycolysis. Similar to our observations in the context of LatA exposure (Figure 2), Zuin *et al.* (2005) demonstrate that Pap1p translocates from the cytoplasm to the nucleus in the presence of high concentrations of the metabolite. Furthermore, they show that this translocation is independent of changes in the redox state of Pap1p. Taken together, these data clearly demonstrate that: (1) Pap1p can be made to enter the nucleus in response to distinct upstream signals, and (2) that these signals need not be related to oxidative stress.

In addition to responding to multiple upstream signaling events, Pap1p is also capable of regulating distinct sets of transcriptional targets. For example, the set of Pap1p-dependent genes activated in response to oxidative stress is distinguishable from the set called upon during nitrosative stress (Biswas and Ghosh 2015). This result suggests that Pap1p is used in a modular fashion to link (or map) diverse upstream signals to distinct sets of transcriptional targets. Such a modular role is also supported by our own data, which show that LatA-mediated induction of the *caf5* gene occurs in the absence of concomitant *trr1* induction (indicating, in contrast to the oxidative stress response, that only the multidrug resistance arm of the pathway is activated) (Figure 5).

The fact that Pap1p overexpression confers DNA damage resistance in checkpoint-deficient cells provides a further example of the multifaceted nature of Pap1p function (Belfield *et al.* 2014). Intriguingly, this resistance is observed not only when damage is induced with the use of drugs, but also when it is induced by mutation. Thus, in this instance, resistance cannot simply be explained by the induction of membrane-associated efflux pumps. This suggests that Pap1p may play a much more sophisticated and nuanced role in fission yeast stress responses than previously thought. In any event, by showing that Pap1p translocates into the nucleus in response to LatA-induced cytoskeletal perturbations (Figure 2), we provide another example of an oxidative stress-independent function, and provide further support for the modular nature of Pap1p-dependent transcriptional regulation.

With respect to Pap1p translocation, we provide strong evidence that the α-importin, Imp1p, is the major determinant (Figure 2B). Moreover, we show that transport into the nucleus is physiologically relevant to the LatA-sensitive phenotypes observed (Figure 3). However, the fact that *pap1*Δ cells are more LatA-sensitive than *imp1*Δ mutants implies that another importer is involved (*i.e.*, if Imp1p were solely responsible for Pap1p import, then one would expect *imp1*Δ and *pap1*Δ cells to exhibit the same sensitivity) (Figure 1A). A likely candidate for this second importer is the α-importin, Cut15p. This is suggested by the fact that *cut15-85* temperature-sensitive mutants, while able to accumulate Pap1p in the nucleus in response to hydrogen peroxide treatment, are unable to do so to the same extent as wild-type cells (Umeda *et al.* 2005). Thus, *imp1*Δ mutants may accumulate at least some Pap1p in the nucleus (at low, undetectable levels). This low level of Pap1p might thereby confer moderately increased resistance on *imp1*Δ cells (relative to *pap1*Δ mutants). A similar argument can be used to explain the increased resistance of *caf5*Δ cells when compared to either *pap1*Δ or *imp1*Δ mutants (Figure 1A). This is to say, we suggest that Caf5p is not the sole effector (*i.e.*, transcriptional target) of Pap1p with respect to LatA-mediated cytoskeletal perturbation, and that these other effectors provide some protection from LatA in *caf5*Δ backgrounds.

That fission yeast cells actively respond to LatA-induced damage is supported, not only by the present data (Figure 2 and Figure 5), but by expression profiling experiments demonstrating large scale changes in the expression of core environmental stress response genes in response to low dose treatment with the drug (Saberianfar *et al.* 2011; Rentas *et al.* 2012). The particular molecular event(s) being sensed (and how this information is transduced to Pap1p) remain unknown, but must be downstream of LatA binding to actin. This is to say, a LatA-sensitive, actin-independent event is ruled out by our finding that Pap1p fails to accumulate in the nucleus, or induce *caf5* expression, in *act1-R183A*,*D184A* backgrounds (Figure 4 and Figure 5).

Interestingly, a recent genome-wide screen has shown that, in addition to *pap1*, the function of at least 38 other genes is required to maintain viability in the face of LatA treatment (Asadi *et al.* 2016). Importantly, the main detriment caused by LatA exposure seems to be that of a reduced capacity to successfully complete cytokinesis (*i.e.*, the LatA-sensitive mutants identified in the study invariably display a terminal phenotype characterized by fragmented septa and the inability to complete cell division). This same terminal phenotype is exhibited by *pap1*Δ, *imp1*Δ, and *caf5*Δ mutants indicating that: (1) the dynamic process of cytokinesis is particularly vulnerable to LatA-mediated perturbation, and (2) that Pap1p-mediated induction of *caf5* (and potentially other as yet unidentified targets) is sufficient to negate the effects of the drug and allow constriction of the actomyosin ring (Figure 1B). However, the molecular functions of Pap1p, Imp1p, and Caf5p suggest that any role in cytokinesis is almost certainly indirect.

In conclusion, it is interesting to note recent work suggesting a role for actin as a cellular biosensor. As pointed out by Smethurst *et al.* (2014), the actin cytoskeleton is acutely sensitive to a variety of stresses and, at least in budding yeast, is centrally positioned as part of a series of signal transduction pathways that monitor environmental change (*e.g.*, the cAMP/PKA pathway, pheromone response pathway, cell wall integrity pathway, and TOR pathway). Thus, it is perhaps not surprising that actin dynamics might be monitored and used as a signal to effect large scale transcriptional changes that aid in adaptation to the initiating stress and which promote cell survival and proliferation. In this respect, it is interesting to speculate as to the potential role of other stress responsive pathways (*e.g.*, the Spc1p/Sty1p pathway) in integrating upstream signals to modulate the selectivity of the Pap1p-dependent transcriptional response.

## Supplementary Material

Supplemental material is available online at www.g3journal.org/lookup/suppl/doi:10.1534/g3.116.037903/-/DC1.
