# Supplementary figures and images for: Latrunculin A-Induced Perturbation of the Actin Cytoskeleton Mediates Pap1p-Dependent Induction of the Caf5p Efflux Pump in Schizosaccharomyces pombe
Source: G3 (Bethesda). 2016 Dec 29;7(2):723–30. doi: 10.1534/g3.116.037903 (PMC5295615; doi:10.1534/g3.116.037903)

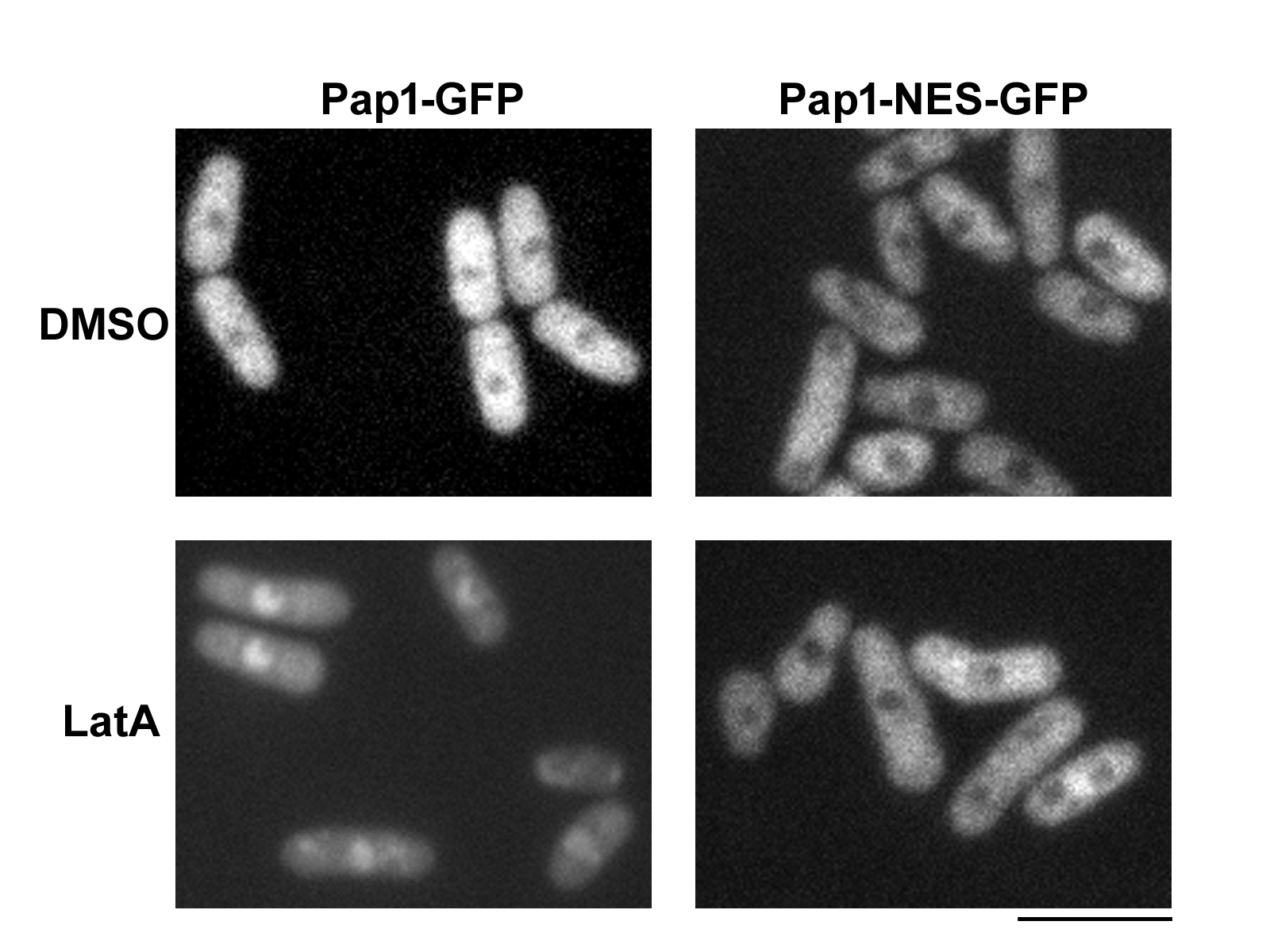

Supplement: Supplementary file 1 [file 723FigureS1.tif]

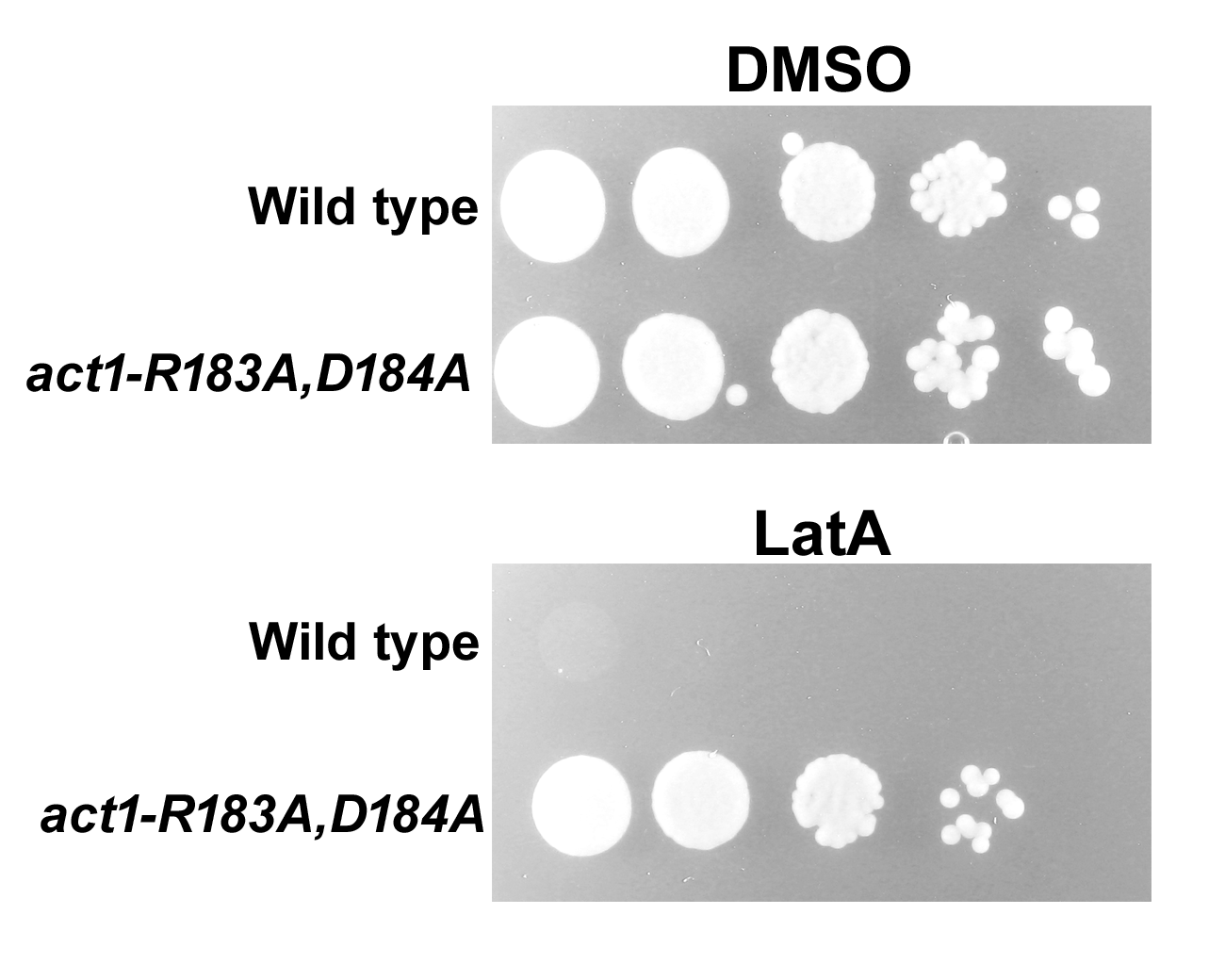

Supplement: Supplementary file 2 [file 723FigureS2.tif]

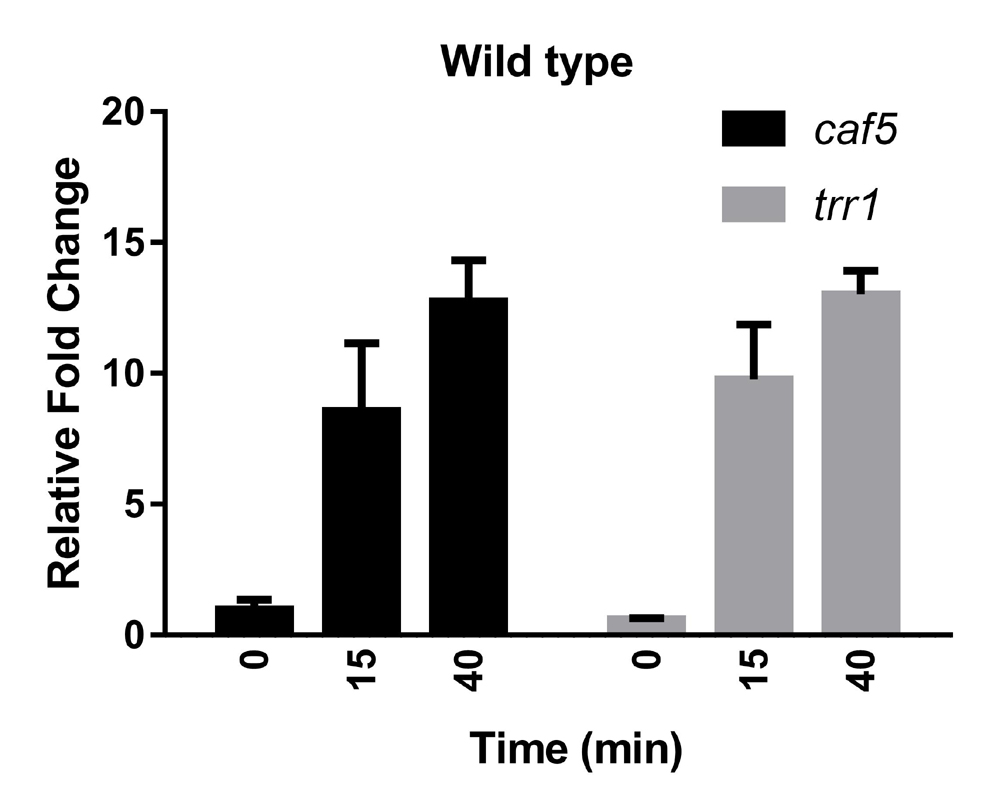

Supplement: Supplementary file 3 [file 723FigureS3.tif]
